# Upgrading the quality of Africa's rice: a novel artisanal parboiling technology for rice processors in sub‐Saharan Africa

**DOI:** 10.1002/fsn3.242

**Published:** 2015-05-22

**Authors:** Sali Atanga Ndindeng, John Manful, Koichi Futakuchi, Delphine Mapiemfu‐Lamare, Joséphine M. Akoa‐Etoa, Erasmus N. Tang, Jude Bigoga, Seth Graham‐Acquaah, Jean Moreira

**Affiliations:** ^1^ AfricaRice Center01 BP 2031CotonouBenin; ^2^ Institute of Agricultural Research for Development (IRAD)BP 2123YaoundéCameroon; ^3^ Department of BiochemistryFaculty of ScienceUniversity of Yaoundé‐IYaoundéCameroon

**Keywords:** Cooking properties, equipment efficiency, parboiling, physicochemical properties, quality, rice

## Abstract

In order to increase the quality of locally produced rice, the artisanal parboiling process in West and Central Africa was reconceptualized. A novel parboiling unit was constructed using stainless steel (Inox 304) and fitted directly on an improved stove made from fired bricks. The heat profile at different locations in the unit, the physicochemical properties, cooking properties of the parboiled rice, and the fuel efficiency of the stove were evaluated and compared with that of the traditional system. The heat flow in the new unit was from the top to the bottom while the reverse occurred in the traditional unit. The percent impurities and heat‐damaged grains, swelling and water uptake ratios, amylose content, stickiness, and cohesiveness were lower for rice produced using the improved technology (IT) compared to the traditional technology (TT). Whole grains (%), lightness (L*), yellowness (b*), cooking time, viscosity were higher for rice produced using the IT compared to the TT. Most of physicochemical and cooking properties of rice produced using the IT were not different from that of premium quality imported rice and this was achieved when steaming time was between 20–25 min. The improved stove recorded a lower time to boil water and specific fuel consumption and a higher burning rate and firepower at the hot‐start high‐power phase compared to the traditional stove. Most end users rated the IT as easy and safe to use compared to the TT. The new technology was code‐named “Grain quality enhancer, Energy‐efficient and durable Material (GEM) parboiling technology.”

## Introduction

Rice (*Oryza* spp.) is fast becoming a staple in West and Central Africa although local demand far exceeds production with most countries relying on imports to meet local demand (IRRI 2012). Although farm production is on the rise, qualitative and quantitative postharvest losses along the rice value chain have remained very high due to poor postharvest practices. Physical losses from the farm to the table are estimated at 30% (IRRI 2015). In addition, there are significant qualitative losses as locally produced rice is less attractive (high percentage of broken fractions, chalky grains, and impurities) and sold mostly unbranded (Demont et al. 2012; Ndindeng et al. 2014). Efforts to reduce postharvest losses to <10% will significantly increase available rice on the market and it is believed that this action alone will in the short term have a greater impact on increasing the availability of locally produced rice (Ndindeng et al. 2014). The lower quality of local rice vis‐à‐vis imported rice does not allow it to compete effectively with imported rice in urban markets in West and Central Africa (Demont et al. 2012; Demont 2013) and this has had a negative effect on investments in the region's rice sector. Preference for imported rice in the raw (uncooked) state appears to be common across Africa especially in urban centers, so matching the quality of imported rice can be an excellent target for the improvement of locally produced rice (Futakuchi et al. 2013).

Rice parboiling if properly carried out, improves significantly the physical, eating and nutritional quality of the milled rice compared to the nonparboiled counterpart (Bhattacharya 1985; Manful et al. 2009; Odenigbo et al. 2013). Rice parboiling thus provides an opportunity for the transformation of poor quality paddy from farmer's fields that may be due to changing climatic conditions and poor farmer‐miller practices in sub‐Saharan Africa (SSA) countries to good quality milled rice (Ndindeng et al. 2014). Parboiled rice is rice that has its starch partially gelatinized by soaking paddy rice or brown rice in water followed by steaming and a drying process. Parboiling increases grain translucency and decreases chalkiness due to starch pregelatinization (Bhattacharya 1969). It also increases grain hardness and manages to reduce grain breakage as a result of the swelling of the starchy endosperm during gelatinization, which heals the preexisting defects (Rao and Juliano 1970; Bhattacharya 1985). Milled parboiled rice has also been reported to have a lower glycemic index, higher resistant starch content, and higher contents of B‐vitamins than milled nonparboiled rice (Jenkins et al. 1988; Newton et al. 2011; Odenigbo et al. 2013). In addition, parboiled rice has been shown to possess some unique cooking, flavor, and textural characteristics that are appealing to certain groups of consumers (Heinemann et al. 2006; Prom‐U‐thai et al. 2010; Demont et al. 2012).

In West and Central Africa, artisanal rice parboiling (hand processing under atmospheric pressure) is common due to its high demand and this is predominated by women. The quantity of paddy parboiled varies from 15 to 100 kg per batch using diverse rudimentary equipment and procedures. The quality of milled parboiled rice in SSA is generally low both in physical (darker color, high % broken fractions, impurities, heat‐damaged grains) and eating (highly cohesive) quality. Studies on artisanal parboiling have focused on the effect of soaking temperature and steaming time on some grain quality characteristics (Manful et al. 2009; Graham‐Acquaah et al. 2015) but these have been limited to laboratory scale quantities (300–500 g of rice) making it difficult to extrapolate the results to bigger systems that are common in the field. In addition, few studies have reported on the heat profile in artisanal parboiling units and the fuel efficiency of the stoves as these are critical in producing large quantities of high‐quality parboiled rice under artisanal conditions. In SSA, medium scale (300–1200 kg paddy/day) processors use several cast iron (GG‐10) drums with a false bottom for soaking and steaming with the energy source being a three‐stone fireplace which have been recognized to have lower fuel efficiency and higher carbon monoxide, carbon dioxide and hydrocarbon (C_x_H_y_) emissions in the field (Jetter and Kariher 2009; Yank, A., Ngadi, M., Kok, R., Ndindeng, S.A. unpubl. data). Additionally, the three‐stone fireplaces expose rice processors to heat burns especially during steaming. Recently, a parboiling unit was developed in Benin that gave a better quality product (Houssou and Amonsou 2004) compared to the traditional units. However, the steam conservation and distribution in this unit during steaming were inadequate, as paddy at the bottom, receives more steam than that at the top and this results in rice that is not evenly parboiled. The system also recorded a high amount of heat loss at the sides and at the top. In addition, the material used to produce the steaming vessel (galvanized cast iron) was not appropriate, as oxidation leading to rust was observed after a relatively short period of usage.

Under artisanal parboiling conditions, the soaking temperature for the variety, steaming time, fire intensity, quantity of paddy being steamed, steam conservation, and distribution in the system are important to achieve high‐quality parboiled paddy (Islam et al. 2002; Graham‐Acquaah et al. 2015). In this paper, a novel artisanal rice parboiling technology that relies on a different heat profile as that of the traditional unit is described. The grain quality characteristics of parboiled rice produced using the new unit were compared with those of traditional parboiled, imported premium quality parboiled, and nonparboiled samples. The burning rate, specific fuel consumption, firepower, and turn‐down ratio of the improved stove were compared with that of the traditional stoves. .

## Materials and Methods

### Rice varieties

TOX 3145, an improved rice variety obtained from the Africa Rice Center (AfricaRice) was used for this study. A sample with high proportion of paddy grains having fissures was used to compare the effectiveness of the different parboiling equipments. The presence of fissures in the grains was determined using the image analysis method (Courtois et al. 2010; Ndindeng et al. 2014). This variety is widely cultivated in Cameroon and is the variety of choice for parboiling. It is common to find paddy from farmer's fields with a high proportion of fissures (Ndindeng et al. 2014). Premium quality imported rice branded DOCTHI from Thailand was used as a control.

### Description of the parboiling vessels

**Figure 1:**
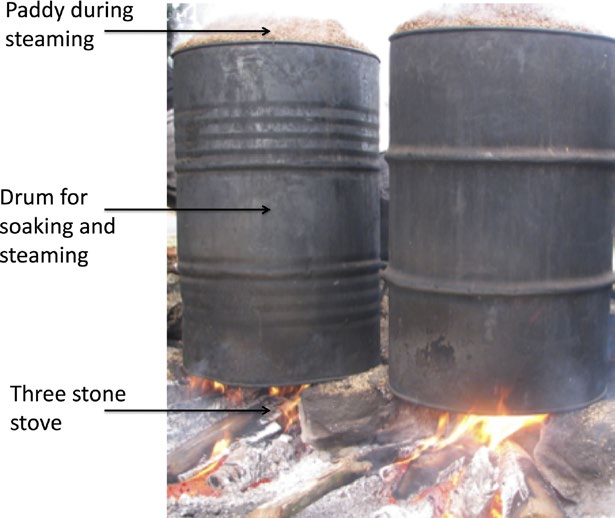
A traditional parboiling unit made of cast iron (GG10) in use during steaming on a 3‐stone stove.

**Figure 2:**
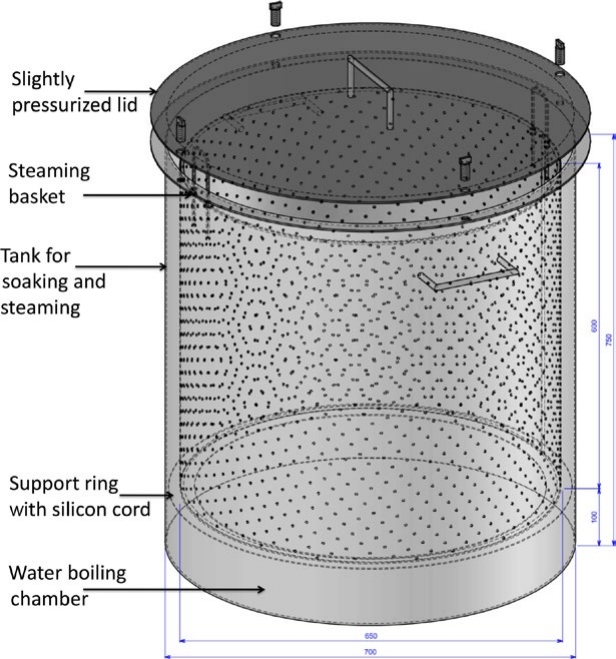
An improved parboiling unit made up of a tank and a steaming basket constructed using stainless steel (Inox 304).

The following parboiling equipments were used: (1) Traditional unit (TU) parboiler, which consisted of a 100‐L cast iron (GG 10) drum for soaking, a perforated cast iron sheet suspended on wood or cast iron blocks called “the separator” that was placed in the drum and holds 100 kg of paddy inside the drum during steaming. The separator created a false bottom that separated the water‐boiling chamber from the steaming chamber. The drums were neither covered during soaking nor steaming (Fig. 1). (2) The improved unit (IU) parboiler consisted of a stainless steel (Inox 304) tank with a cover for soaking and a stainless steel mesh basket for holding the soaked paddy that is placed inside the tank during steaming. The tank was closed using a tight‐fitting lid, which is not completely pressurized (Fig. 2). The complete AutoCAD drawings of the assembled IU, tank, basket and lid are shown in Supporting information in Fig. S1, S2, S3 and S4 respectively.

### Description of the energy systems for parboiling

**Figure 3:**
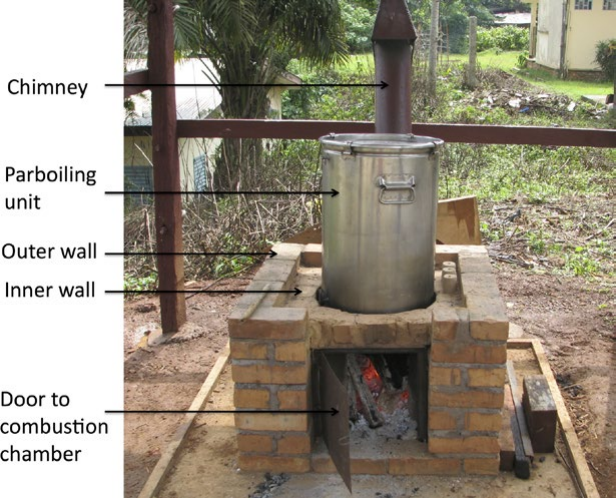
An improved parboiling technology composed of an improved parboiling unit fitted directly on an improved stove made from fired bricks.

The energy systems used for parboiling were: (1) the traditional stove (TS), which consisted of three stones placed in a triangular arrangement (three‐stone fireplace). Wood was fed on one side of the triangle or on all the sides (Fig. 1) and (2) an improved stove (IS), designed by the Local Material Promotion Authority (MIPROMALO) Yaoundé‐Cameroon, was adapted to allow the bottom of the GEM parboiling unit to fit perfectly on top of the combustion chamber, thus reducing heat loss (Fig. 3). This stove was built on a concrete floor. The floor of the stove was made of a single layer of locally produced fired bricks and the walls made of two layers of fired bricks surrounding the cylindrical combustion chamber. The fired bricks were made using clay that was rich in kaolin and could sustain intense heat of up to 1200°C (Vaughan 1955). A “door” leading into the combustion chamber to put wood, charcoal, or rice husk briquettes remained slightly opened during combustion to allow air into the combustion chamber. A chimney was placed at a position that was 3/4 the height of the combustion chamber to ensure the flow of air through the combustion chamber.

### Description of the parboiling process

#### Paddy cleaning

Paddy was cleaned using a mechanical winnower and further washed with water. Floating grains were collected into a separate container. The good quality paddy was carefully transferred into a clean container and care was taken to exclude any sand and gravel that may have settled at the bottom of the washing container.

#### Paddy soaking

Water was added to the paddy at a ratio of 2:1 on weight basis and heated to 80°C using the respective soaking vessels for each parboiling system. The paddy was then left under ambient conditions in the vessels for 16 h.

#### Steaming of soaked paddy

The soaked paddy was collected and transferred into the respective steaming vessels and steamed using vapor generated within the steaming vessels. The steaming time for each vessel was recorded when the husks of grains on top and in the middle of the vessels had split open.

#### Drying

Parboiled paddy (30% moisture content wet basis) was sun‐dried to 18% moisture content. Drying was continued in the shade to 14% moisture content. Drying was done on tarpaulin placed on elevated cemented surfaces with 3 kg/m^2^ drying density. The paddy was tempered overnight and moisture content was checked to ensure that it was between 12–14% before milling.

#### Milling

Rice samples were dehusked using a large‐scale AGRINDO^®^ Rice Huller (P.T. Agrindo, Driyorejo, Indonesia) and polished using a large‐scale SB10D rubber roll mill (Satake‐Corporation, Hiroshima, Japan).

### Heat profile in the vessel during steaming

**Figure 4:**
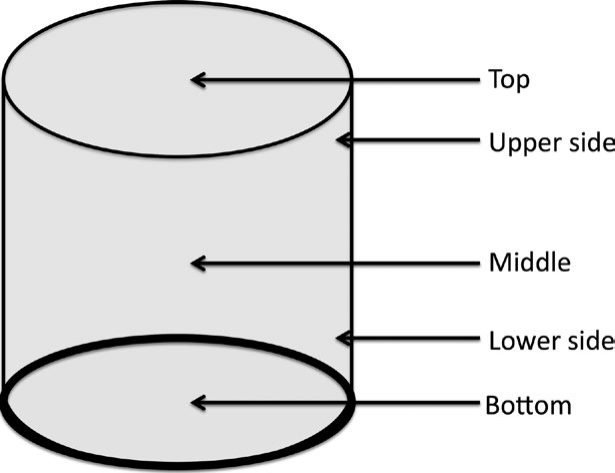
Location of thermal probes in parboiling unit for the determination of heat profile in each unit.

Thermal probes were inserted at the bottom, lower sides, middle, upper side, and top of the steaming vessel (Fig. 4) and the temperatures were recorded every 5 min during steaming using Traceable^®^ Expanded‐Range thermometer (Control Company, Texas, TX, USA) to determine heat profile in the vessel during steaming. Since the probes are wires connected to the hand‐held recorder, the wires were held in the assigned location by the paddy. The experiment was stopped when the temperature in all the locations was ≥100°C. The heat profile was compared in a cold system (paddy inserted in tank before water for steaming is heated to boiling point) and hot system (paddy inserted when water for steaming is already boiling). Two sets of vessels were used for each type of treatment and the experiment was replicated three times.

### Grain quality analysis

The grain quality parameters were carried out in the AfricaRice grain quality laboratory. The number of replicates for each parameter was three. The main parameters evaluated were given below.

#### Physical quality

##### Whole grains (%)

For each sample, 200 g of milled rice was separated into whole and broken grains using a Testing Rice Grader (Satake‐Corporation) The weight of whole grains was expressed as a percentage of the 200 g used.

##### Impurities and heat‐damaged grains (%)

The percent impurities and heat‐damaged grains were evaluated from a sample of 100 g by observing grains on a blue background. The percent heat‐damaged grains and impurities were the weight in grams of those grains recorded from the 100 g analyzed.

##### Color of grains

The color of grains was measured with a color meter (CR‐400, Minolta Co., Ltd., Tokyo, Japan) using the L*, a*, b* uniform color space procedure. The value of L* expresses the psychometric lightness value, and a* and b* are factors expressing hue and saturation of the color intensity. Seven (7) grams of samples were placed in the sampling cup and the mean L* and b* values were recorded after three shots. Lightness (L*) and yellowness (b*) were the values of interest as they have been identified as the most important indicators of parboiled rice color (Islam et al. 2002; Patindol et al. 2008).

##### Chalkiness (%)

Chalkiness was estimated as described by Graham‐Acquaah et al. (2015) using a Rice Statistical Analyzer (Technologia S21 LKL, Santa Cruz do Rio Pardo, Brazil). Approximately 50 g of whole grains was weighed and emptied into its sample receiver. The “long white” classification set up for the raw nonparboiled rice and the “parboiled rice” classification set up for the parboiled samples were opened in the capture mode on the software. The equipment was then switched on to vibrate and cause the release of individual grains from the receiver to slide on a blue tile background and pass beneath the attached camera that captured images of the grains. When all the grains had exited the receiver, the image‐capturing mode was stopped. The chalkiness of the samples was then estimated by processing the captured images and applying the “basic filter – chalky distribution” on the software. The percentage of total chalky area for the sample was then recorded and reported as the percentage chalkiness of the sample.

#### Chemical quality

##### Apparent amylose content (%)

Apparent amylose content was measured using the standard iodine colorimetric method ISO 6647‐2‐2011 (Juliano 1971). Ethanol (1 mL, 95%) and 1 mol/L sodium hydroxide (9 mL) was added to rice flour (100 mg) and this was heated in a boiling water bath until gelatinization of the starch occurred. After cooling, 1 mol/L acetic acid (1 mL) and iodine solution (2 mL) were added and the volume made up to 100 mL with Millipore water. The iodine solution was prepared by dissolving 0.2 g iodine and 2.0 g potassium iodide in 100 mL Millipore water. Absorbance of the solution was measured using an Auto Analyzer 3 (Seal Analytical, Noderderstedt, Germany) at 600 nm. Apparent amylose content was quantified from a standard curve generated from absorbance values of four well‐known standard rice varieties (IR65, IR24, IR64, and IR8).

##### Pasting properties

The pasting properties of rice flour samples were determined using a Rapid Visco Analyzer (RVA) model‐ super4 (Newport Scientific, Warriewood, NSW, Australia) and Thermocline for Windows (TCW3) software. The general pasting method 162 (ICC 2004) for flour samples was used. Rice flour (3 g sample) was weighed directly into the RVA canister; 25 mL of distilled water was added and mixed with the rice flour. The canister and its content were then place in the RVA and run using the following RVA test profile:

1. Rotating paddle speed – 160 rpm,
2. Heating the rice‐water mixture to 95°C at a rate of 12°C/min (i.e., in 3.75 min),
3. Holding at 95°C for 2.5 min,
4. Cooling to 50°C at a rate of 12°C/min (i.e., in 3.75 min), and
5. Holding at 50°C for 2.5 min.

#### Cooking quality

##### Cooking time

Cooking time was determined using the method described by Fofana et al. (2011). Five grams of milled rice of each sample were poured into 135 mL of vigorously boiling distilled water in a 400‐mL beaker and covered with a watch glass. After 10 min of further boiling, 10 grains were taken out every minute with a perforated ladle. The grains were pressed between two petri dishes and were considered cooked when at least nine out of the 10 grains no longer had opaque centers. The time it took for this to happen was then recorded as the optimum cooking time for the sample.

##### Water uptake and Swelling ratios

The swelling and water uptake ratios were determined according to the method of Fofana et al. (2011). Briefly, eight grams (8 g) of whole milled rice of each sample was put into a wire mesh basket. The weight and the height of the raw rice in the cooking basket were measured using a vernier caliper. The samples were then cooked using the predetermined optimum cooking times of the samples. The cooking basket was subsequently removed and stood erect for the water to drain off. The weight and the height of the cooked rice in the cooking basket were measured and the water uptake and swelling ratio calculated.

##### Texture profile analysis

The texture of samples cooked using the cooking time determined above was analyzed using a TA.XT2 Universal texture analyzer (Stable Micro Systems Ltd, Surrey, UK). The texture profile analysis (TPA) settings were set as follows; speed set = 5 mm/sec, target mode = distance, distance = 25 mm, time = 5 sec, probe = P/35. A force distance curve was obtained from the test and the following textural parameters were determined: hardness = peak force of the first compression cycle, stickiness = area of the negative force curve, representing the work to separate the plunger from the sample on the upstroke after the first curve, cohesiveness = ratio of area under second compression to area under first compression (Champagne et al. 1998). The hardness, stickiness, and cohesiveness of the samples are reported.

### Thermal metrics of parboiling stoves

The water boiling test (WBT) was used to compare the energy efficiency of the traditional and improved parboiling stoves. The WBT protocol version 4.1.2 (Bailis et al. 2009) that consisted of three phases, the cold‐start high‐power test, the hot‐start high‐power test, and the simmer test were performed successively on each stove type using the same type of wood and pots. The data were entered into the *WBT_data‐calculation‐sheet_4.1.2_updated_19‐Jun‐2012* downloaded from (http://www.aprovecho.org/lab/pubs/testing). The following parameters were compared; time to boil water, burning rate, specific fuel consumption, fire power during all the phases of the WBT, and turndown ratio during the simmering phase.

### User‐friendliness of parboiling systems

Forty‐ (40) processors who regularly parboil rice using the TU from two groups (Ngoketunjia Rice Miller's Union and Federation of Integrated Farmers Ndop) in Ndop, Northwest region of Cameroon were asked to use the 20 kg model of the IU to produce parboiled rice. Later they were asked to rate the both systems with respect to easy‐to‐handle and easy‐to‐get‐burn‐injury on a 5‐point hedonic scale (very easy, easy, neutral, not easy, not very easy).

### Statistical analysis

The data obtained were analyzed using the Statistical Package for Social Sciences (SPSS version 10.1.4; 1998) at a 5% significance level. For each grain quality and fuel efficiency parameter, the mean ± standard deviation was computed and reported. The least significant difference (LSD) was used to compare the grain quality characteristics of rice samples among the different parboiling treatments, while the Student's *t*‐test was used to compare the fuel efficiency parameters of the stoves.

## Results and Discussions

### Improved parboiling technology

The improved technology was made up of two components: an improved unit (IU) parboiler used for soaking and steaming that fitted on an improved stove (IS) made from fired bricks. The IU parboiler allowed for several soaking batches to be done and transferred into stand‐by container. In addition, since the vessel generating the steam (tank) was different from that holding the paddy during steaming (mesh basket), several steaming batches were done using the same water already producing steam in the tank (Fig. 2). This made it possible to up‐scale the quantity of parboiled rice being produced and reduced water and energy wastage. All these were not possible while using the traditional unit (TU) parboiler. Although batch soaking and steaming allowed for the scaling up of the quantity of paddy to be parboiled, this largely depended on the availability of containers to accommodate the paddy after soaking, labor, energy, water, and drying surface for the parboiled paddy. Loss of steam was very low in the IU parboiler as the steaming basket sat inside the tank during steaming and the lid fitted perfectly on the tank although not completely pressurized. Very little amount of steam was seen escaping from the top only after 20–25 min of steaming and this time coincided with the minimum and maximum steaming time. In the traditional unit, steam loss was high as large amounts of steam were seen escaping from the top of the unit since the drum comes without a lid. Steaming time thus ranged from 51 to 78 min in the TU parboiler (data not shown in the tables and figures).

The parboiling stove that was constructed with fired bricks on which the parboiling unit fitted reduced heat loss and the chances of excessive heat exposure to the processors during operation (Fig. 3). In addition, the time to boil 5 L of water, burning rate, specific fuel consumption, firepower, and turndown ratio for the improved stove were better (*P* < 0.05) than for the traditional stove, especially at the hot‐start high‐power and simmering phases confirming earlier studies by Miah et al. (2009). The above results show that the IU parboiler was fuel‐efficient as heat loss is reduced compared to the TU parboiler. The firepower of any stove depends on the specific fuel consumption, with a higher amount of fuel consumption expected to yield higher firepower (Jetter and Kariher 2009; Miah et al. 2009). However, this was not the case with the traditional stove (TS) where higher fuel consumption and lower firepower were observed compared to the IS especially at the hot start and simmering phases (Table 1) demonstrating lower fuel efficiency (Jetter and Kariher 2009). The nature of the TS allowed for a high amount of heat loss and irrespective of the amount of fuel added, the steaming time did not stay within the range of 20–25 min for larger quantities of paddy (100 kg) (data not shown in Tables and Figures).

**Table 1:** The fuel efficiency of two stoves used for parboiling determined using the water‐boiling test

Parboiling stove	Phases of the water‐boiling test	Time to boil 5 liters of water (min)	Burning rate (g/min)	Specific fuel consumption (g/liters)	Firepower (watts)	Turndown ratio
Traditional	Cold‐start high power	15.06 ± 0.03	78.53 ± 0.20	487.47 ± 0.26	23889.43 ± 0.26	–
Improved		10.06 ± 0.03*	208.46 ± 0.26*	884.47 ± 0.26*	63723.60 ± 0.30*	–
Traditional	Hot‐start high power	14.10 ± 0.50	204.56 ± 0.20	1119.50 ± 0.28*	62532.50 ± 0.28	–
Improved		08.06 ± 0.03*	259.96 ± 0.54*	879.40 ± 0.10	79653.50 ± 0.28*	–
Traditional	Simmering	–	41.50 ± 0.26	18451.80 ± 0.15*	12584.50 ± 0.26	1.90 ± 0.12
Improved		–	92.60 ± 0.30*	16746.33 ± 0.28	28187.46 ± 0.26*	2.24 ± 0.05*

*Implies significant difference of the means during each phase of the water‐boiling test at the 0.05 level of significance using student t‐test.

Although a detailed cost‐benefit analysis is in‐progress for this technology, it is important to indicate that the IU parboiler costs $1500 and has a life span of about 10 years since the materials used was stainless steel (Inox 304). In comparison, three cast iron drums (CG‐10) needed to parboil a similar quantity of paddy per day costs $100 but need to be replaced every 3 months. This brings the cost of the TPU to $4300 over a period of 10 years.

### Heat profile in the vessel during steaming

**Figure 5:**
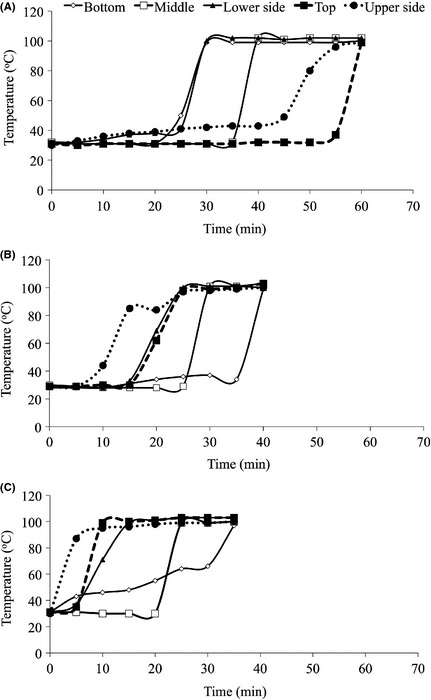
Heat flow in the (A) Traditional unit, (B) improved unit when paddy is introduced in a cold system (not producing steam) (C) an improved unit when paddy is introduced in a hot system (already producing steam).

The heat profile in the IU parboiler was different from that in the TU parboiler currently in use in the region. Heat flow in the TU parboiler was from the bottom, through the sides to the middle before reaching the top (Fig. 5A). In the IU parboiler, heat flow was from the top and upper sides through the lower sides and middle to the bottom (Fig. 5B). The heat flow in the system was generally better when the paddy was introduced in a hot system than when it was introduced in a cold system (Fig. 5B and C). This was the recommended practice in the IU parboiler but this is rather difficult to implement in the TU parboiler, as there was need for water and steamed paddy to be removed from the container before the next batch of steaming could be initiated using the same vessel.

### Physico‐chemical and cooking quality of parboiled rice

The different types of treatments influenced the physicochemical and cooking quality of the rice. Improved parboiled rice (IPR) and premium imported parboiled rice (PIPR) were comparable (*P* > 0.05) with respect to levels of impurities, heat‐damaged grains, whole grains, color, swelling ratio, paste set back viscosity, and stickiness (Table 2). The percent impurities and heat‐damaged grains, swelling and water uptake ratios, apparent amylose content, stickiness, and cohesiveness were lower for IPR compared to TPR. Meanwhile whole grains, lightness, yellowness, cooking time, viscosity were higher for IPR compared to TPR.

**Table 2:** The physicochemical and cooking quality of rice following different processing treatments

Sample	Impurities (%)	Heat‐damaged grains (%)	Whole grain (%)	Lightness (L*)	Yellowness (b*)	Cooking time (min)	Swelling ratio	Water uptake ratio
TPR	6.04 ± 2.96^a^ *	23.9 ± 4.66^a^	53.66 ± 6.07^b^	45.51 ± 0.09^c^	13.55 ± 0.08^c^	18.5 ± 0.70^b^	2.93 ± 0.15^a^	1.30 ± 0.05^b^
GPR	0.43 ± 0.00^b^	6.19 ± 0.84^b^	91.47 ± 1.82^a^	54.23 ± 0.03^b^	14.72 ± 0.01^b^	20.5 ± 0.70^a^	2.52 ± 0.06^b^	1.13 ± 0.05^c^
PIPR (Imported)	0.00 ± 0.00^b^	2.94 ± 1.08^b^	94.97 ± 1.17^a^	53.36 ± 0.54^b^	15.21 ± 0.01^a^	19.0 ± 0.00^b^	2.45 ± 0.12^b^	1.41 ± 0.04^b^
NPR	3.83 ± 0.49^a^	NA	38.27 ± 0.29^c^	66.07 ± 0.29^a^	11.77 ± 0.01^d^	17.0 ± 0.00^c^	2.84 ± 0.05^a^	1.69 ± 0.02^a^

Sample	Chalky grains (%)	Apparent amylose content (%)	Peak viscosity (cP)	Final viscosity (cP)	Setback viscosity (cP)	Texture Profile Analysis		
						Hardness (kg)	Stickiness (N.s)	Cohesiveness
TPR	0.00 ± 0.00^bb^	23.700 ± 0.38^b^	120.5 ± 04.94^d^	185.0 ± 07.07^d^	64.5 ± 02.12^c^	6.01 ± 0.55^a^	−0.003 ± 0.001^a^	0.58 ± 0.028^a^
IPR	0.00 ± 0.00^bb^	20.822 ± 0.55^c^	924.5 ± 09.19^b^	1565.5 ± 26.16^b^	641.0 ± 16.97^b^	6.00 ± 0.34^a^	−0.010 ± 0.008^b^	0.52 ± 0.006^b^
PIPR	0.06 ± 0.08^b^	19.891 ± 1.21^c^	646.0 ± 02.82^c^	1289.5 ± 09.19^c^	643.5 ± 12.02^b^	5.05 ± 0.49^b^	−0.012 ± 0.002^b^	0.53 ± 0.008^b^
NPR	7.34 ± 2.80^a^	28.681 ± 0.29^a^	2818.5 ± 48.79^a^	5318.0 ± 31.11^a^	2499.5 ± 79.90^a^	4.91 ± 0.44^b^	−0.006 ± 0.003^a^	0.50 ± 0.020^b^

TPR, traditional parboiled rice; IPR, improved parboiled rice; PIPR, premium imported parboiled rice; NPR, nonparboiled rice; NA, not applicable.

*Means with different superscript letters implies least significant difference at the 0.05 level of significance using one‐way Analysis of variance.

The percent whole grain was higher in all parboiled samples compared to their nonparboiled counterpart. This result is consistent with earlier studies where parboiling was shown to reduce grain breakage as a result of the swelling of the starchy endosperm during gelatinization, which heals the preexisting defects (Rao and Juliano 1970; Kato et al. 1983; Bhattacharya 1985). However, amongst the parboiled samples, percent whole grains for nongraded IPR was comparable to that of PIPR (*P* > 0.05) and higher than TPR (*P* < 0.05). These results indicated that gelatinization was fully achieved in the IPR sample. The nonuniform distribution of steam and longer steaming time in the TU parboiler may be the main reason for this difference as portions not properly exposed to steam may have resulted in high amount of broken grains. Patindol et al. (2008) reported that nonuniform or partial parboiled rice was more susceptible to breakage on milling. Partially parboiled grains were observed in the TPR sample.

The percent heat‐damaged grains for IPR and PIPR were comparable and lower than that for TPR (*P* < 0.05). Longer steaming times for example have been shown to increase the level of heat‐damaged grains in parboiled rice (Bhattacharya 1985). This was probably responsible for high heat‐damaged grains recorded with TPR where longer steaming time was recorded. Under atmospheric pressure, the higher the fire intensity (firepower), the shorter the steaming time since the temperature in the steaming vessel will be higher. High steaming time resulted to high percent heat‐damaged grains especially at the bottom and lower sides of the TU parboiler. A steaming time of 60 min for example produced on average 60% heat‐damaged grains from the bottom and lower sides of the TU parboiler (data not shown in tables and figures). When the steaming time in the IU parboiler was 20 min, the average percentage of heat‐damaged grains was 1.33%. Increasing the time to 25 and 40 min also resulted in an increase in heat‐damaged grains to 6.19% and 26.71%, respectively. After 60 min of steaming, the percentage heat‐damaged grains were 26.71, 33.73, and 30.53 in samples collected at the bottom, sides, and middle‐top, respectively, of the IU parboiler. For the TU parboiler, it was 51.66, 68.33, and 34.33, respectively, (data not shown in table and figures). These results demonstrated that heat conservation and distribution in the IU parboiler were better than in the TU parboiler as samples collected at different locations in the IU parboiler at 60 min of steaming showed similar amount of heat damage grains compared to those collected from different location in the TU parboiler. In the IU parboiler, the paddy in the steaming basket was placed inside the tank during steaming; this system gave better uniformly parboiled product. The above results suggests that proper adjustments need to be made on the quantity of paddy per batch being steamed, heat conservation, and distribution in the steaming unit and the firepower of the stove during steaming to ensure that steaming is completed within 20–25 min otherwise the percentage of heat‐damaged grains will be increased substantially in the sample. It is worth noting that when steaming time was also <15 min, parboiling was incomplete resulting in high levels of broken fractions and chalky grains.

Although the overall goal is to produce whiter parboiled rice, parboiling treatment generally discolors grains and reduces the lightness (L*) value (Ali and Ojha 1976; Islam et al. 2002). Longer steaming time and higher steaming temperature have also been shown to reduce lightness in parboiled rice (Bhattacharya 1969; Islam et al. 2002). The L* values of IPR and PIPR were comparable and higher than that of TPR (*P* < 0.05). Amongst the parboiled samples, PIPR had the highest yellowness (b*) value while TPR had the least. Parboiled rice turns light yellow to amber due to Maillard type nonenzymatic browning and the diffusion of the husk and bran pigments into the endosperm during soaking (Bhattacharya 2004; Lamberts et al. 2006).

Parboiling eliminates chalkiness and increases grain translucency (Manful et al. 2008). Low chalky score generally indicates better sensory quality both for nonparboiled and parboiled rice (Kim et al. 2000). All parboiled samples in this study had very low chalky scores. Although TPR had no chalky grains, the absence of chalky areas did not necessarily imply good quality for this rice type.

Parboiling increases the cooking time compared to nonparboiled rice. It took a longer time to cook IPR than for PIPR and TPR that had comparable cooking time.

Parboiling reduces the apparent amylose content of rice compared to the nonparboiled counterpart (Patindol et al. 2008). IPR and PIPR had comparable apparent amylose content and it was lower than that of TPR (*P* < 0.05). The decrease in apparent amylose content has been attributed to leaching during soaking or measurement errors owing to interference by milled rice surface lipids (Patindol et al. 2008). Proper parboiled rice will have a high amount of lipids bound to starch (Kato et al. 1983) and this may explain why IPR recorded lower apparent amylose content compared to TPR.

The slurry viscosities (peak, final, and setback) were higher in nonparboiled samples than in parboiled samples (*P* < 0.05) as observed by other studies (Unnikrishnan and Bhattacharya 1981; Patindol et al. 2008). However, TPR had lower peak, final, and setback viscosity compared to the IPR and PIPR (*P* < 0.05) which this was comparable indicating that swelling of starch granules was retarded in the TPR than in the IPR and PIPR (Unnikrishnan and Bhattacharya 1981; Patindol et al. 2008).

The swelling ratio of cooked:uncooked grains for IPR and PIPR was comparable and lower than that for TPR and NPR. These findings are in line with work by Unnikrishnan and Bhattacharya (1981) that showed that parboiled rice flour swelled less than nonparboiled rice flour at higher temperatures (>70°C) and this was similar to what was experienced in whole grains. This result might be due to the high amount of partially parboiled grains in the TPR sample.

The hardness of cooked parboiled rice was higher than that of the nonparboiled counterpart and these results are consistent with previous findings (Kato et al. 1983; Islam et al. 2002; Patindol et al. 2008). The hardness of cooked IPR was comparable to that of TPR and both were lower than that of PIPR.

IPR and PIPR showed similar level of stickiness (*P* > 0.05) and this was lower than the TPR and NPR (*P* < 0.05). Parboiling generally reduces stickiness (Kato et al. 1983; Patindol et al. 2008) but this was not the case with TPR that showed the same level of stickiness as NPR (*P* > 0.05).

The cohesiveness of IPR, PIPR, and NPR were comparable (*P* > 0.05) and lower than that of TPR suggesting differences in sensory attributes. Consumers prefer rice that is less cohesive (Kato et al. 1983) suggesting high cohesiveness of locally parboiled rice is one of the factors negatively affecting consumer preference of locally parboiled rice. This has recently been confirmed in framed market experiments where Akoa Etoa et al. (2015) demonstrated that the majority of the participants (63%) could not distinguish between the IPR and the PIPR type. In addition, interviewees were prepared to pay $ 0.15/kg less for TPR and $ 0.12/kg more for the IPR rice. These results show that the major value of the improved parboiling technology is that it produces parboiled rice whose quality mimics that of premium quality imported rice. Those who thought the IPR was imported selected the following attributes to describe the quality of that sample; clean, high percent whole grains, attractive color, not sticky, and swells better after cooking (Akoa Etoa et al. 2015).

Both artisanal and industrial processes are used to produce parboiled rice. Industrial processes are energy and capital intensive and are not suitable for small to medium scale operations at the village level (Roy et al. 2006). Under artisanal parboiling conditions, the soaking temperature for the variety, fire intensity, quantity of paddy, steam conservation, and distribution in the system are important to achieving high‐quality parboiled rice. These conditions must be set to allow for steaming to be completed in 20–25 min after the water starts to produce steam. Steaming times that are longer will allow for some grains to be over steamed resulting in complete cooking rather than being precooked (parboiled). Higher than optimal soaking temperatures for a given variety and overexposure of some grains to steam are the main causes of heat‐damaged grains and nonuniformity in parboiled rice observed in West and Central Africa. Sorting to get rid of discolored grains and impurities using different types of rice color sorters is a common practice in industrial parboiling plants. However, at the village level in SSA, this is done manually by handpicking the discolored grains and the levels may be high in some samples (>20%). During the course of this study, it was observed that handpicking of discolored grains was laborious, time consuming, and costly to the processor ($ 0.05/kg in Benin).

The values obtained for impurities, heat‐damaged grains, whole grains, lightness, swelling ratio, set back viscosity, and stickiness for IPR and PIPR can be used as reference values to check the quality of artisanal parboiled rice in SSA countries. Parboiled rice with characteristic values different from those indicated, as was the case with TPR, might suggest poorer quality and this may be due to the rudimentary nature of the equipment and/or the processing procedures followed. The differences observed in these properties were linked to the steaming time, which was 20–25 min for the IU parboiler and 51–78 min for the TU parboiler. Islam et al. (2002) recommended a steaming time of 20 or 30 min for parboiling when the steaming temperature was 100 or 90°C. Soaking temperature and steaming time have been shown to affect the physicochemical and cooking properties of parboiled rice (Graham‐Acquaah et al. 2015).

### User‐friendliness of the improved parboiling technology

Thirty‐six (36) processors who used the IU parboiler in Ndop rated the unit as easy‐to‐handle compared to only four who said so for the TU parboiler. The size, weight, and content of the paddy holding mesh basket (20 kg) during steaming made it easy to be lifted out of the tank to the drying surface. All the processors said it was not very easy to get burn injuries when using the improved parboiling technology, especially if the operator was not standing at the side holding the chimney. On the contrary, all participants rated the traditional system as very easy to get burn injuries while in use. These results show that the improved parboiling technology was user‐friendly. In addition, soaking and steaming with the stainless steel material used to construct the IU were advantageous, as the material had the required strength, corrosion resistance, formable, and weldable (Partington et al. 2007) compared to the cast iron used to build the TU.

## Conclusion

This study describes for the first time an artisanal parboiling technology that was reconceptualized to improve the distribution of steam and reduce heat loss during parboiling. The new parboiling unit was constructed using stainless steel (Inox 304) and fitted directly on an improved stove made from fired bricks. This technology was code‐named “Grain quality enhancer, Energy‐efficient and durable Material (GEM) parboiling technology.” The heat flow in the GEM unit was from the top to the bottom, while the reverse occurred in the traditional unit. The percent impurities and heat‐damaged grains, swelling and water uptake ratios, apparent amylose content, stickiness, and cohesiveness were lower for rice produced using the GEM technology compared to the traditional technology (TT). Whole grains (%), lightness (L*), yellowness (b*), cooking time, viscosity were higher for rice produced using the GEM technology compared to the TT. Most of physicochemical and cooking properties of rice produced using the GEM technology were not different from that of premium quality imported rice and this was achieved when steaming time was between 20–25 min. The improved stove recorded a lower time to boil water and specific fuel consumption and a higher burning rate and firepower at the hot‐start high‐power phase compared to the traditional 3‐stone stove. Most end users rated the GEM technology as easy and safe to use compared to the TT.

The values of impurities, heat‐damaged grains, whole grains, lightness, swelling ratio, paste set back viscosity, and stickiness for premium quality imported parboiled rice and GEM parboiled rice could be used as reference values for assessing good quality parboiled rice under artisanal parboiling conditions in the region. The GEM parboiling technology is easy to build and up‐scale at the village level. It is worth noting that bigger GEM systems (50, 65 and 100 kg) have been developed and end user evaluations are in progress and will be reported in due course. This is likely to increase the chances of these technologies being adopted by small to medium scale rice processors that are in the majority in SSA and as such increase the economic benefits and disposable incomes of rice value‐chain actors in the region.

## Conflict of Interest

The authors do not have any conflict of interest.

## Supporting information


**Figure S1.** Drawing of the assembled improved unit parboiler.
**Figure S2.** Drawing of the soaking and steaming tank.
**Figure S3.** Drawing of the steaming basket.
**Figure S4.** Drawing of the tight‐fitting lid.
