# Supplementary material for: Upgrading the quality of Africa's rice: a novel artisanal parboiling technology for rice processors in sub‐Saharan Africa
Source: Food Sci Nutr. 2015 May 22;3(6):557–68. doi: 10.1002/fsn3.242 (PMC4708646; doi:10.1002/fsn3.242)
Supplement: Supplementary file 1 — Figure S1. Drawing of the assembled improved unit parboiler. Figure S2. Drawing of the soaking and steaming tank. Figure S3. Drawing of the steaming basket. Figure S4. Drawing of the tight‐fitting lid. [file FSN3-3-557-s001.pdf]

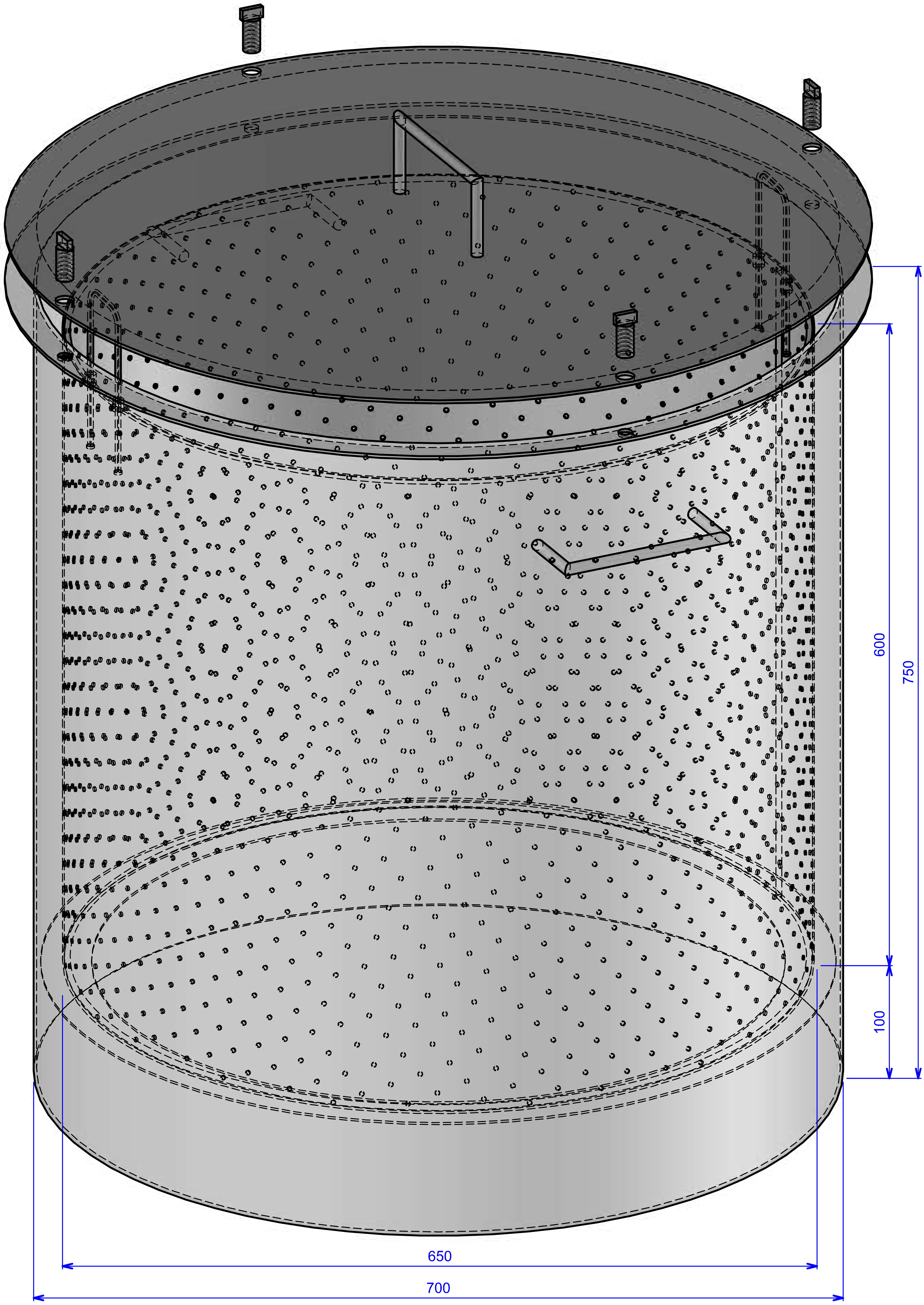

SCALE 3 :10	GEM PARBOILING UNIT	NUMBER 1<br>DATE 15/09/2014
A3		01

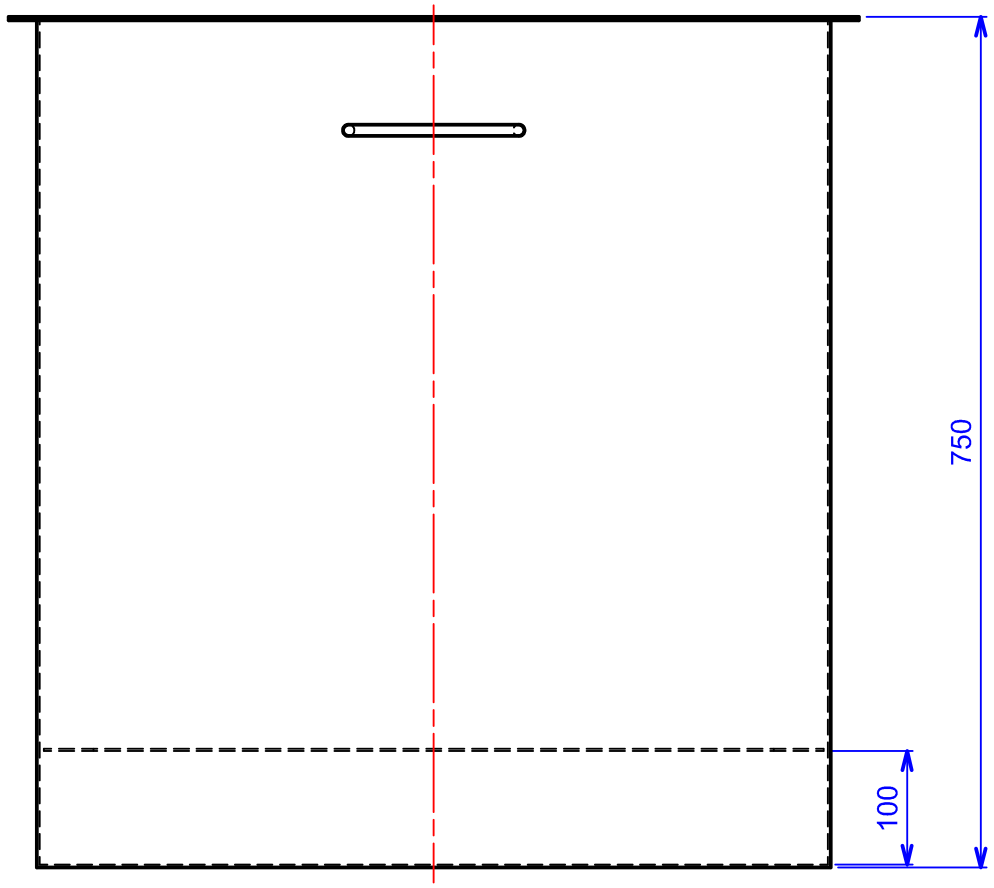

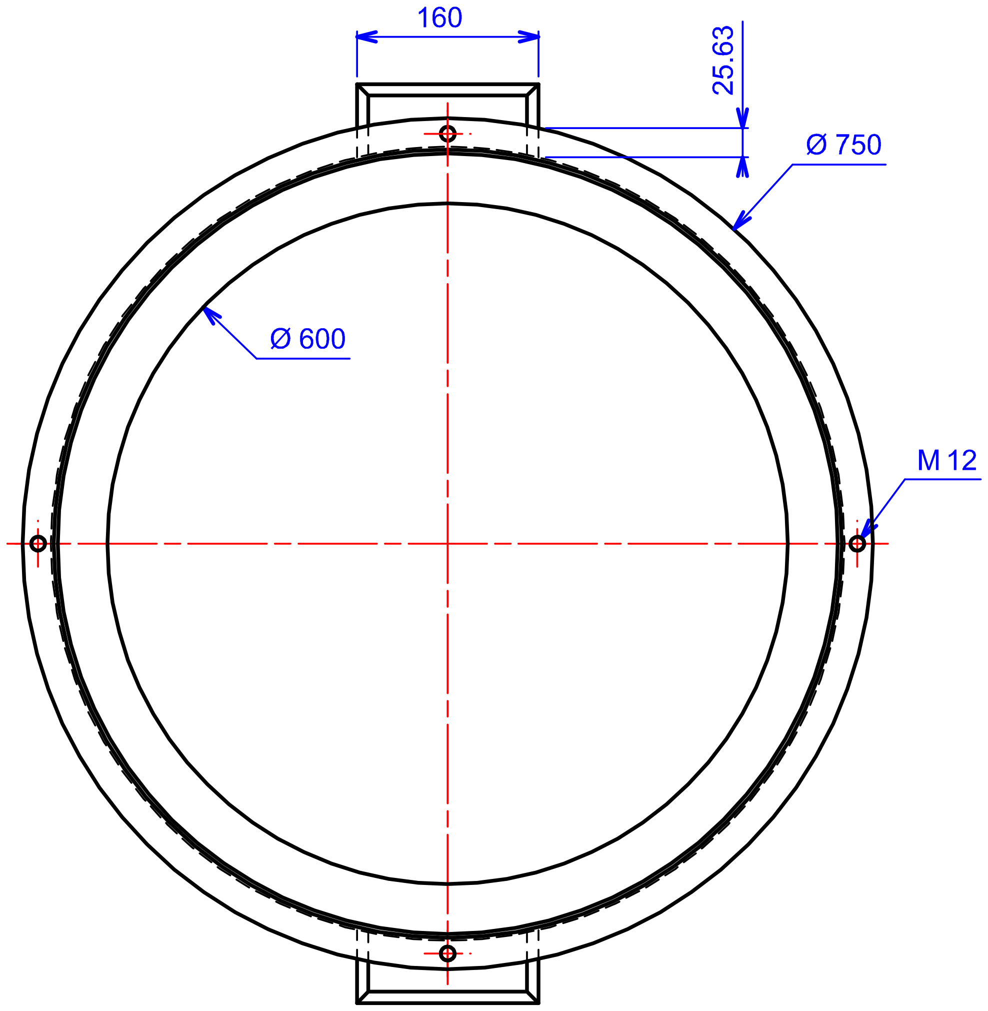

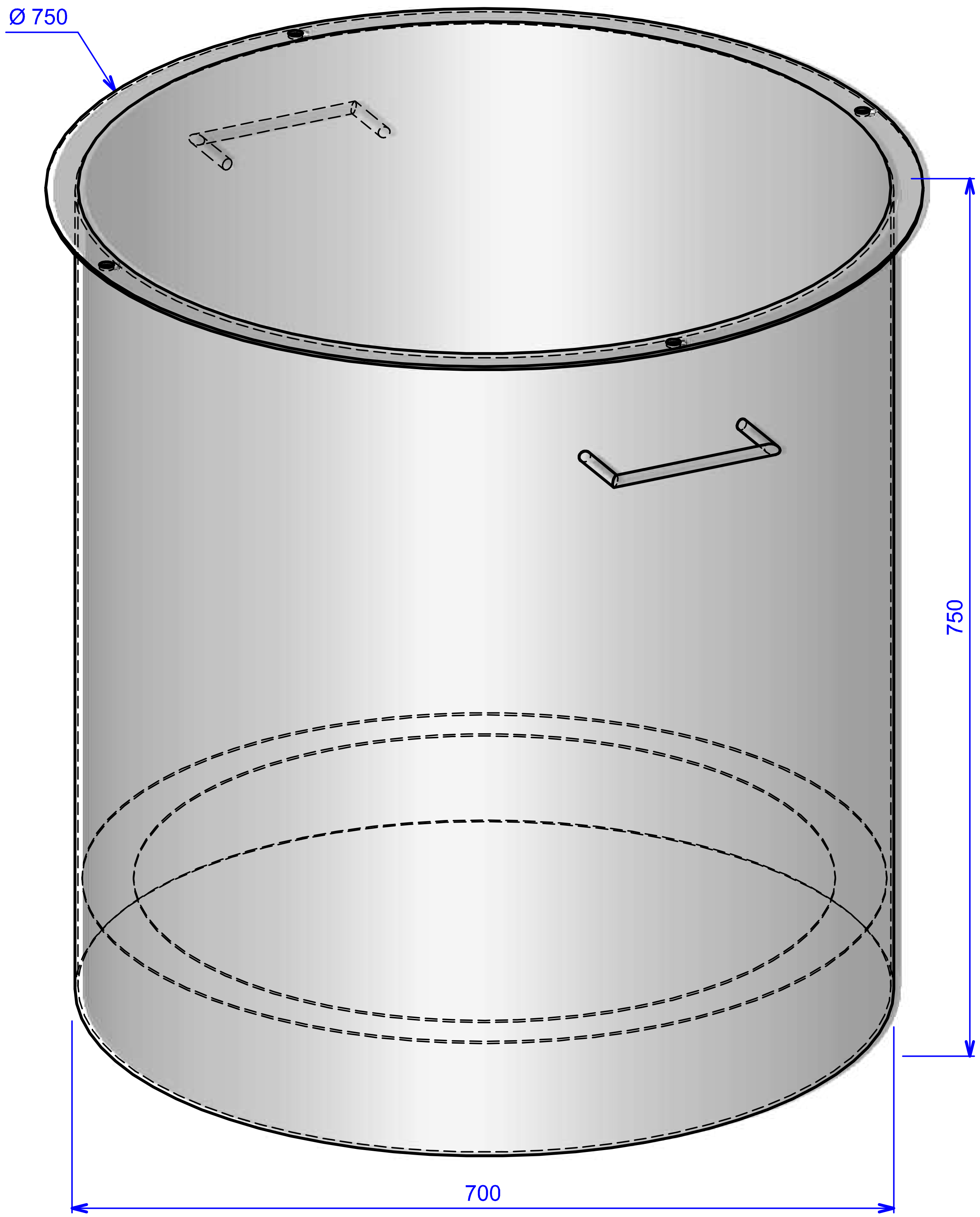

SCALE	TANK FOR SOAKING AND STEAMING	NUMBER 2
15 :10<br>2 :10		DATE<br>15/09/2014
A3		02

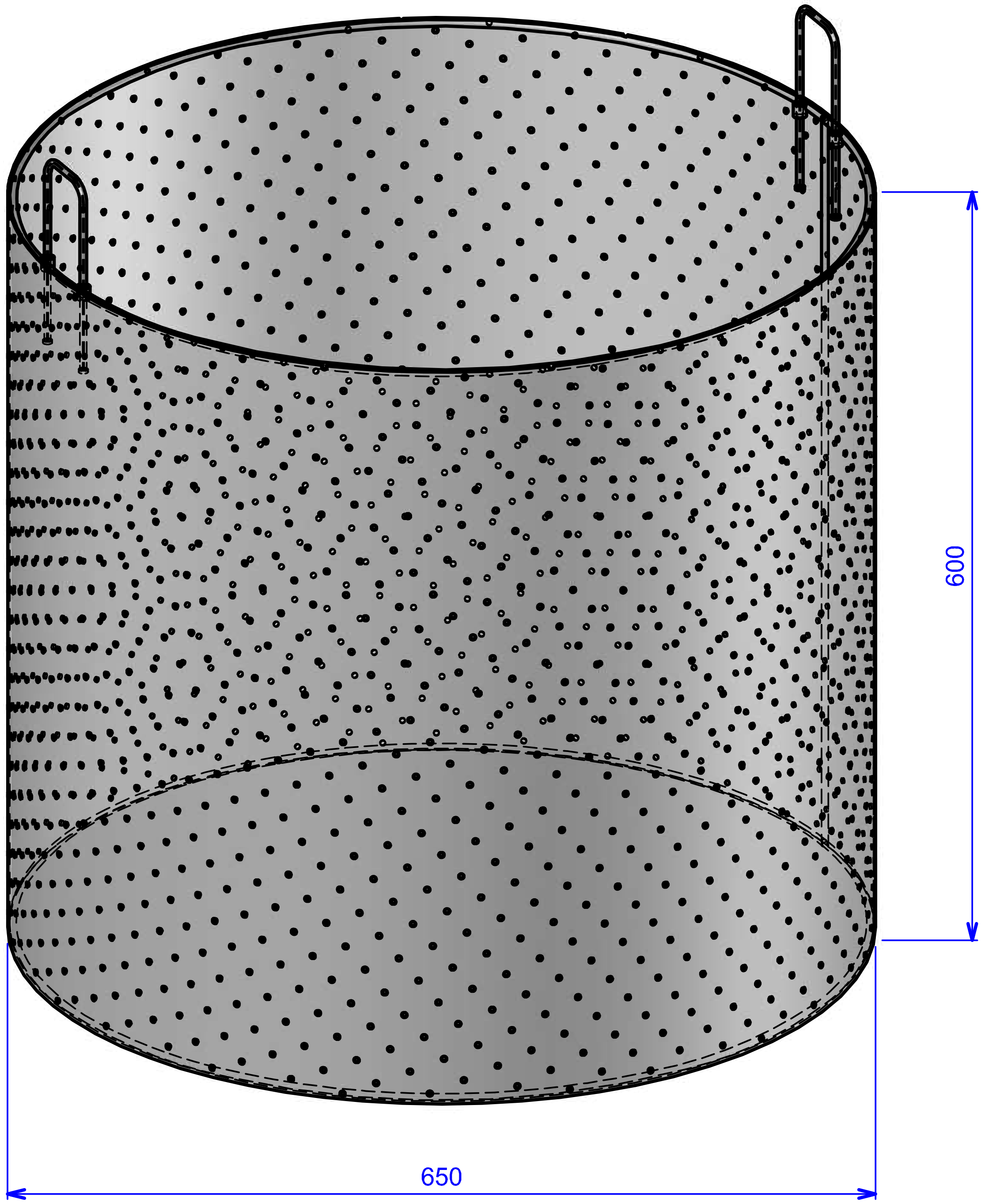

SCALE 2 :10	BASKET	NUMBER 3
		DATE 15/09/2014
A4		03

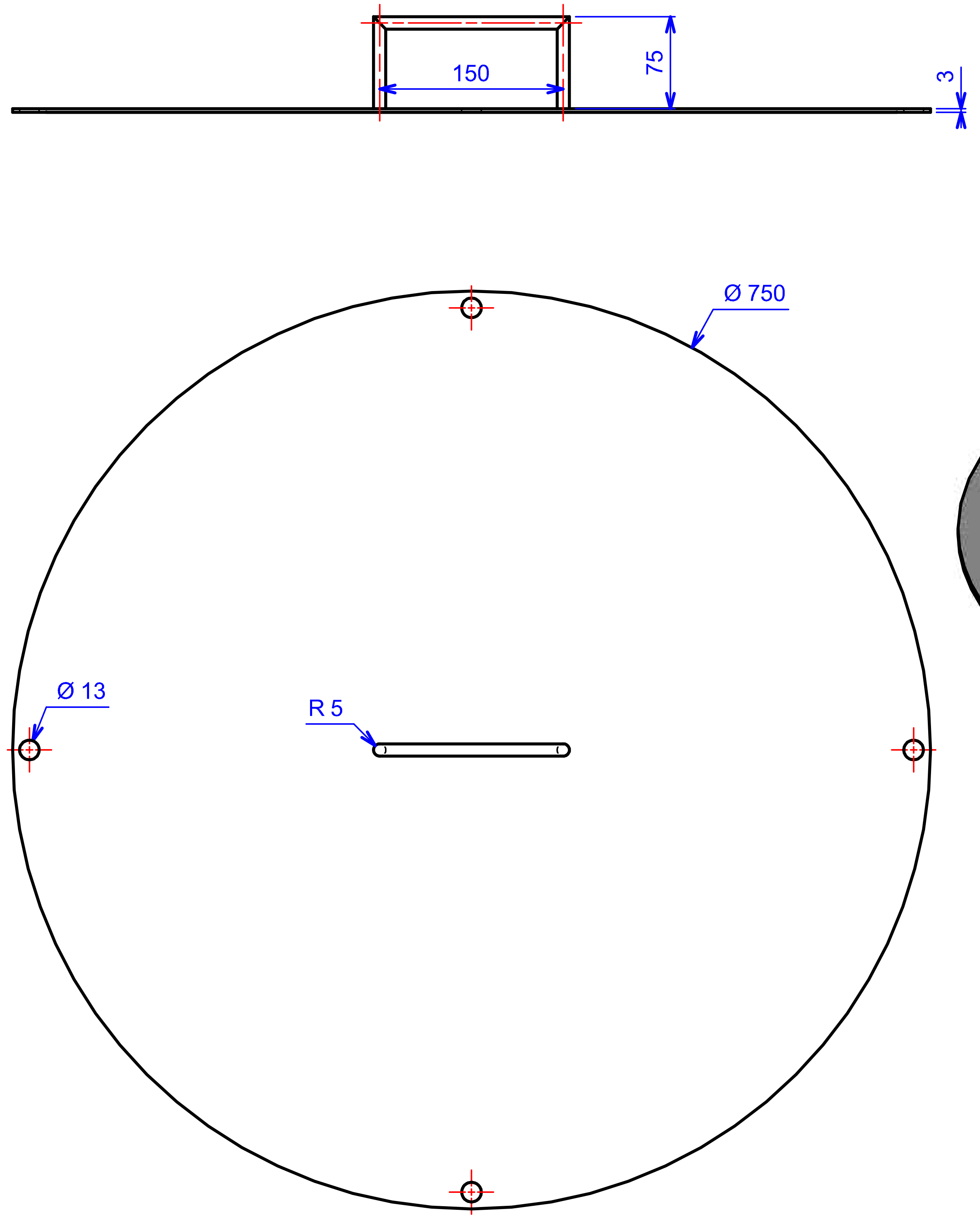

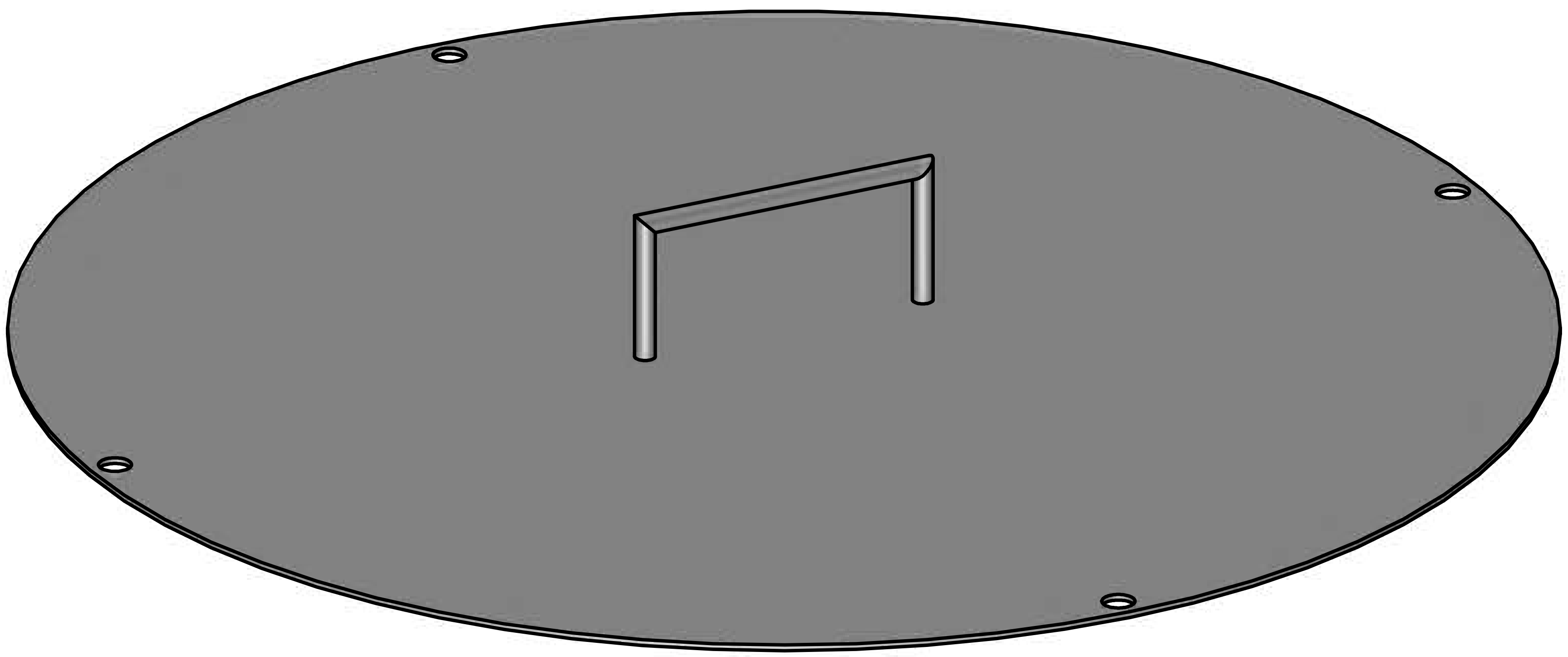

SCALE 3 :10	HEAVY LID	NUMBER 4	
		DATE 15/09/2014	
A3			04
